# The Relationship Between Religious/Spiritual Beliefs and Subjective Well-Being: A Case-Based Comparative Cross-National Study

**DOI:** 10.1007/s10943-022-01550-4

**Published:** 2022-04-17

**Authors:** Sergio Pérez, Daniela Rohde

**Affiliations:** 1 School of Social Work and Social Policy, Trinity College Dublin, Dublin, Republic of Ireland; 2 Present Address: Department of Medical Gerontology, TILDA, School of Medicine, Trinity College Dublin, Dublin, Republic of Ireland

**Keywords:** Religious beliefs, Supernatural beliefs, Subjective well-being, Terror management theory, Cross-national comparisons

## Abstract

The most unique aspect of religiosity/spirituality (R/S), supernatural beliefs, and their relationship with SWB has hardly been examined. This study explores the relationship between six R/S supernatural beliefs and SWB, in a case-based comparative cross-national design including two religious and two secular nations. Data were obtained from the International Social Survey Programme (ISSP) Religion IV module from the religious countries of the USA (*n* = 1060) and Turkey (*n* = 1353) and the secular countries of Denmark (*n* = 1281) and Czech Republic (*n* = 1112). SWB was measured as happiness and self-rated health. Statistical analyses were performed using binary logistic regression models replicated across countries. Results indicated that the American sample showed no evidence of relationships between R/S and SWB outcomes capable of improving the model over demographic and service attendance covariates. In Turkey, some R/S beliefs were found to be statistically significantly related to SWB, with positive and negative associations with happiness. No associations were found in the secular countries. Findings were discussed in the light of previous research and interpreted from a terror management theory perspective.

## Introduction

In the last few decades, there has been a growing interest in the study of religion and spirituality in relation to many health and psychological aspects. In particular, attention has been drawn to the question of whether religion and spirituality can be beneficial for people’s general health and subjective well-being (SWB). This is an important question as almost 90% of the world population adheres to some religious faith (Johnson & Grim, 2013), and most people see SWB as an important part of their lives (Maddux, 2018). The most prominent researchers in this area of study have suggested that religiosity and spirituality (R/S) are positively linked to health and SWB (Hood et al., 2009; Koenig et al., 2012; Krause et al., 2019a; Pargament, 2002) with some authors being confident enough to argue for a cause and effect relationship (Oman, 2018; Oman & Syme, 2018). The purported benefits of R/S on SWB have also been implicitly generalised, with the assumption that religiosity is a universal part of the being human (Kier & Davenport, 2004). However, criticisms of this research have highlighted methodological problems, cultural and researcher biases, and an overwhelming majority of studies coming from the USA alone (Belzen, 2004; Cragun et al., 2016; Hwang et al., 2011; Sloan & Bagiella, 2002).

Moreover, religion and spirituality have been defined in multiple and sometimes obscure ways, and mostly assessed through single item self-reported measures, such as church attendance, that lack construct validity (Flannelly, 2017; Hwang et al., 2011). Here, we adopt the definition of religion offered by Bruce (2011), who states that religion consists of ‘*beliefs, actions and institutions which assume the existence of supernatural entities with powers of action, or impersonal powers or processes possessed of moral purpose*’ (p. 112, emphasis in original). Spirituality, on the other hand, can be generally described as belief in God (or a higher power) as well as the subjective experience of having a relationship with this supernatural entity (Zinnbauer et al., 1997). It should be noted that both definitions share an emphasis on the supernatural, and spirituality has been associated with religiosity throughout history (Koenig et al., 2012). Given this conceptual and historical overlap, here both concepts are used in combination under the abbreviation ‘R/S’ (Hwang et al., 2011; Koenig et al., 2012). SWB is an overarching construct that incorporates the person’s positive and negative emotional responses (an emotional component), and the person’s own judgement of his/her life satisfaction in general and on specific domains (a cognitive component) (Diener et al., 1999).

### Shifting the Focus from R/S to R/S Beliefs

In spite of the body of research devoted to exploring the R/S and SWB association, a considerable gap remains in terms of investigating the intrinsic and unique element of R/S that could be related to SWB. Instead, research has concentrated on identifying possible pathways to explain the relationship between the two, proposing social support, health behaviours, psychological resources, and a sense of meaning as possible explanatory mechanisms (George et al., 2002; Oman & Thoresen, 2005). These pathways are useful to understand how R/S may exert an indirect effect on health and SWB through mediating mechanisms. However, none of these pathways reflect anything intrinsically or exclusively religious or spiritual, that is, the benefits of those mechanisms can also be accrued through secular means (Cragun et al., 2016; Galen, 2017; Galen & Kloet, 2011; Kier & Davenport, 2004). For example, social support can be found in many other social groups apart from church congregations, such as community centres, sport clubs, universities, and workplaces (Cragun et al., 2016).

Therefore, if one is to investigate the relationship between R/S and SWB, and wants to get at the heart of the matter, why not focus on whether the unique aspects of R/S are linked to SWB? And what would these unique aspects be? It has been argued that beliefs are the most fundamental component of R/S (Krause, 2010a; Stark & Glock, 1968), and that the supernatural is the defining feature of R/S (Routledge, 2018; Thrower, 1980; Zuckerman, 2020). (Note this is in line with the definitions of religion and spirituality previously offered.) We propose then, as some researchers have already done, that when exploring relationships between R/S and SWB, the focus be placed on supernatural R/S *beliefs*. Indeed, Lun and Bond (2013) advocated for the use of more exhaustive measures of R/S and SWB, recommending the inclusion of ‘measures about an individual’s belief in the existence of a supernatural world’ in future research (p.10).

### R/S Beliefs and SWB: Overview of the Literature

In comparison with the extensive literature on R/S and SWB in general, there is only a small number of studies that specifically focus on the relationship between R/S beliefs and SWB (Flannelly, 2017). Here, we review some of the most common R/S beliefs (in Abrahamic religions) that have received attention: feelings of attachment to God, the afterlife, heaven, hell, and evil forces (Argyle, 2005; Park, 2017).

The belief of being close to and having a relationship with God has been explored by studies of religious attachment, extending classical attachment theory to attachment to God (ATG) (Granqvist, 2014). Leman et al. (2018) found that secure ATG uniquely predicted self-reported psychological health (explaining approximately 4% of the variance) after controlling for other religious variables. Positive effects of secure ATG on psychological well-being were also found in studies controlling for other types of adult attachment (Keefer & Brown, 2018; Njus & Scharmer, 2020). Longitudinal studies, however, have shown mixed results regarding the benefits of secure ATG on life satisfaction (Bradshaw & Kent, 2018; Ellison et al., 2012).

Belief in an afterlife is common among religious people and highly prevalent in the USA and in countries with Catholic majorities (Flannelly, 2017). Afterlife beliefs have been found to be positively associated with life satisfaction (Cohen et al., 2005), well-being (Ellison et al., 2001), and feelings of tranquillity (Ellison et al., 2009). Flannelly et al. (2008) analysed the relationship between belief in an afterlife and severity of a number of psychiatric symptoms and found that while pleasant afterlife beliefs (e.g. reunion with loved ones) were negatively associated with severity of psychiatric symptoms, unpleasant beliefs (e.g. ‘a pale, shadowy form of life’) indicated a detrimental impact on mental health. Shariff and Aknin (2014) examined the association between beliefs in heaven and hell and SWB. The study comprised a cross-national comparison of 63 countries, and measured life satisfaction and daily affect (SWB), beliefs in heaven and hell, general religiosity, and a number of socioeconomic control variables at a national level (e.g. unemployment, GINI index, GDP). The results showed that beliefs in heaven and hell predicted higher and lower levels of SWB, respectively. Notably, these two variables were the strongest predictors in the regression model, making contributions that surpassed GDP and unemployment.

Beliefs about evil supernatural forces, the devil, or demons, are part of many religions. In the USA, 58%, 56%, and 48% of Americans ‘absolutely’ believe in Satan, hell, and demons, respectively (Baker, 2008). Nie and Olson (2016) examined longitudinal data from a national American survey on religion and young people and found that belief in demons predicted lower levels of mental health in the later waves. In another study, belief in Satan was also found to be positively associated with a number of psychiatric symptoms, particularly with paranoid ideation (Flannelly, 2017).

### Cultural Context and Terror Management Theory

In addition to exploring the possible relationship between specific R/S beliefs and SWB, a point must be made on the importance of considering the cultural context in this area of research. Studies have shown that religious people appear to enjoy higher levels of SWB in those societies where religiosity is desirable and represents the social norm. In these societies, highly religious individuals can reap the benefits of living within the expected cultural norms, for instance by being more accepted, expanding social networks, and enjoying social support. On the other hand, non-religious people tend to experience lower levels of SWB in highly religious societies, but show high levels of SWB in countries that are highly secular. In other words, people benefit from fitting in within their cultures, the ‘person-culture fit’, and this extends to the R/S and well-being relationship (Lun & Bond, 2013; Stavrova, 2015; Stavrova et al., 2013).

Considering this point, Galen (2017) has argued that in order to properly investigate any associations of R/S and well-being, it is paramount to compare religious and secular cultures. Furthermore, we propose that an investigation of the R/S beliefs and SWB relationship, when examined in the context of religious and secular cultures, would be enriched by applying a theoretical framework that allows a meaningful interpretation of results in different sociocultural contexts.

Terror management theory (TMT) has been advanced as a theory of SWB to explain the effects of both R/S beliefs and secular worldviews on mental health and well-being (Solomon & Thompson, 2019; Vail et al., 2010) and it has been already applied by a number of researchers to explore the relationship between R/S and SBW (Cranney, 2013; Hackney & Sanders, 2003; Joshanloo, 2016; Silton et al., 2014). TMT poses that, as the result of our evolved cognitive capacities, humans face the terrifying realisation of the certainty of mortality. According to TMT, people’s fundamental source of anxiety is fear of death, and in order to buffer this anxiety and continue to function in everyday life, people engage in different cultural worldviews that provide permanence, meaning, and control. By being valued members of their cultural worldview, individuals obtain self-esteem that allows them to manage and cope with death anxiety, and achieve ‘symbolic’ immortality (Greenberg et al., 1986). The theory has generated a number of clear and testable hypotheses that have garnered empirical support in research spanning over 30 countries (Cox et al., 2019). This body of empirical research has shown that under experimental conditions, when the salience of mortality is manipulated and enhanced, people cling to their cultural beliefs (including religion) more strongly to buffer anxiety, and people see others who hold different cultural views more negatively (Greenberg et al., 1997; Vail et al., 2010).

Of all cultural worldviews and collective human endeavours that could be used to quench death anxiety religious worldviews may be the most appealing since religion offers an all-encompassing view of human existence, deals with matters that escape scientific disconfirmation, and more importantly, offers the promise of literal immortality (Soenke et al., 2013; Vail et al., 2010). R/S beliefs fulfil important psychological functions that can help individuals to have hope and feel reassured that death is not the end for them or their loved ones. For example, when religious people are reminded of death, they tend to increase their faith in their beliefs (Dechesne et al., 2003; Norenzayan & Hansen, 2006; Schoenrade, 1989), derogate those who challenge those beliefs (Greenberg et al., 1990; Iqbal et al., 2016), and even avoid using religious objects inappropriately (for instance using a crucifix as a hammer) (Greenberg et al., 1995).

In societies where R/S is not prominent, the adoption of secular worldviews provides a viable alternative that fulfils the same psychological functions for SWB. Although secular worldviews cannot offer ‘literal’ immortality, they do provide ample means by which individuals can feel part of a collective project imbuing life with worth, meaning, and symbolic immortality, protecting them from anxiety (Juhl, 2019). A few examples are having children, belonging to a national culture, humanistic views of life, an appreciation for progress and science, and a variety of hobbies and lifestyles (e.g. focusing on sports, music, art, developing a professional career, etc.) (Solomon & Thompson, 2019).

### The Current Study

While a small number of studies have examined the effect of R/S *beliefs* on SWB, there have been, to the best of our knowledge, no studies that examine *several* R/S beliefs simultaneously *and* also compare results across diverse countries in a cross-national design. This study sought to address this gap by examining the relationship between specific R/S beliefs and SWB, and to explore whether this relationship differed across countries with different cultural and religious backgrounds.

In addition, we wish to apply TMT as a theoretical framework, as well as drawing attention to what type of results could support or falsify a TMT interpretation of the findings. It is hypothesised here that R/S beliefs should have some relationship with SWB in those cultures where R/S is prominent, since religion could be argued to be an important part of the sociocultural fabric of those societies. In short, a TMT interpretation will be supported if:

- A relationship appears to exist between R/S beliefs and SWB in religious countries, but not in secular ones.
  On the contrary, TMT would not be supported in any of the following three cases:
- A relationship between R/S beliefs and SWB appears to exist not only in religious countries, but in secular countries as well
- No relationship between R/S beliefs and SWB is found in neither religious nor secular countries
- Less likely, a relationship seems to be present between R/S beliefs and SWB in secular countries, rather than in religious ones.

## Method

The current study used a case-based comparative cross-national design. This type of design takes a small number of countries (cases) that are selected based on given criteria relevant to the research question or aims. The design aims first to understand each country within its own context, rather than taking each country simply as one of many data points where variables are measured. Then, by comparing similarities and differences among the selected countries, explanations are developed to elucidate possible causes of the observed phenomena. Therefore, the aim of comparative cross-national research is to elucidate to what extent given social phenomena can be explained by patterns that are universal and patterns that occur at the specific level of local cultures or countries (De Vaus, 2008).

### Data Source

Data for this study were obtained from the International Social Survey Programme (ISSP) 2018 module ‘Religion IV’ (Muckenhuber et al., 2020). This ISSP module is unique in its kind since it focuses solely on religion, examining religious attitudes, behaviours, and beliefs, past and present religious practices, and religious socialisation among others aspects (Gesis, 2020). The ISSP 2018 Religion IV comprises data from 33 countries and persons included in the surveys were adults from 18 years of age, with some exceptions (e.g. Denmark did not include people over 80 years old). Countries for the present study were selected based on the majority religion of the country, the proportion of atheism in the surveyed population, and whether they could be categorised as ‘traditional’ or as ‘secular-rational’ according to the Inglehart–Welzel Cultural Map (Wvs, 2020). Inglehart and Welzel (2005) classified countries according to the values that their societies tend to endorse; in traditional countries, religion is generally very important, whereas in secular-rational countries it is not. Country characteristics based on the selection criteria are detailed in Table 1. It was decided to select countries within cultures adherent to Abrahamic religions, since questions in the survey have greater emphasis on these types of religious beliefs. Moreover, it has been noted that the response style to surveys can vary quite substantially in Western and Eastern countries (Wierzbiński & Kuźmińska, 2016). Four countries were thus selected for cross-national comparisons: the USA (religious, Christian), Turkey (religious, Muslim), Denmark, and Czech Republic (both secular).

**Table 1:** Country characteristics according to selection criteria

Country	Majority (non)religious group		Percentage of atheists	Inglehart–Welzel cultural map
	Religion	%		
USA	Protestant	47	4.8	Traditional
Turkey	Islam	98	1.7	Traditional
Denmark	Lutheran	74	27.9	Secular-rational
Czech Republic	No religion	66	34.7	Secular-rational

### Measures

#### Dependent Variables

Subjective well-being was assessed through self-reported measures of happiness and self-rated health (SRH). For happiness, participants responded to the question ‘If you were to consider your life in general these days, how happy or unhappy would you say you are, on the whole…’ The four-point scale responses were categorised into two groups, retaining the happiest group (‘very happy’) and combining those who reported being in less happy groups (‘fairly happy’, ‘not very happy’, and ‘not at all happy’). For SRH, the question was ‘In general, would you say your health is…’ followed by a five-point scale with the options: excellent, very good (which were grouped into ‘very good SRH’), good, fair, and poor (grouped into ‘good SRH or less’).

#### Independent Variables

The independent variables consisted of six specific R/S beliefs. Two of these R/S beliefs, related to the concept of God, were taken from two statements from the root question ‘do you agree or disagree with the following?’: ‘There is a God who concerns Himself with every human being personally’ and ‘To me, life is meaningful only because God exists’. For short, these beliefs will be referred to here as belief in a ‘concerned God’ and in ‘God-giving life meaning’, respectively. Each answer consisted of a five-point Likert scale, with responses recoded into the options ‘agree’ (combining agree and strongly agree responses), ‘neither agree nor disagree’ and ‘disagree’ (combining disagree and strongly disagree responses). The remaining R/S beliefs were taken from the question ‘Do you believe in…’ followed by the beliefs: life after death (also referred as afterlife), hell, heaven, and religious miracles. Answers for each belief were on a four-point scale including ‘yes, definitely’, ‘yes, probably’, ‘no, probably not’, and ‘no, definitely not’, and were dichotomised into yes or no.

Demographic variables included: sex, age-group (≤ 37 years, 38–57 years, 58 + years), level of education (post-secondary or secondary or less), employment status (working or not working), and marital status (married/partnered or not married). Self-placement in society was used as a proxy for socioeconomic status, based on the question: ‘In our society there are groups which tend to be towards the top and those that are towards the bottom. Here, we have a scale that runs from top to bottom. Where would you put yourself on this scale?’ The answer presented a 10-point scale (1 being the lowest place in society, and 10 the highest), which were recoded into two categories: from 1 to 7 ‘middle or bottom’, and from 8 to 10 ‘top’.

In addition, religious service attendance, a global measure of religiosity, was included as a control variable, in line with other studies on R/S beliefs (Flannelly, 2017; Nie & Olson, 2016; Shariff & Aknin, 2014). The survey question asked ‘How often do you attend religious services?’ and offered 9 options, which were recoded into ‘regularly’, ‘irregularly’, and ‘never’. A description of each country sample is displayed in Table 2.

**Table 2:** Description of Country Samples

Variable	USA		Turkey		Denmark		Czech Republic	
	*n*	%	*n*	%	*n*	%	*n*	%
Sex (female)	617	58.2	690	51.0	552	43.1	642	57.7
Age group								
Older adults (58 +)	347	32.7	113	8.4	376	29.4	503	45.2
Middle-aged adults (38 to 57)	369	34.8	509	37.6	445	34.7	373	33.5
Younger adults (≤ 37 years)	344	32.5	731	54.0	460	35.9	236	21.2
Education (post-secondary)	378	35.7	283	20.9	815	63.6	240	21.6
Work status (working)	663	62.5	592	43.8	869	67.8	587	52.8
Marital status (married/partnered)	461	43.5	826	61.0	710	55.4	576	51.8
Self-placement in society (top)	243	22.9	324	23.9	357	27.9	101	9.1
Religious service attendance								
Regularly attends to church	381	35.9	930	68.7	52	4.1	99	8.9
Irregularly attends to church	376	35.5	261	19.3	648	50.6	328	29.5
Never attends to church	303	28.6	162	12.0	581	45.4	685	61.6
Belief in a concerned god								
Agree	727	68.6	1,175	86.8	269	21.0	226	20.3
Neither agree nor disagree	152	14.3	47	3.5	176	13.7	212	19.1
Disagree	181	17.1	131	9.7	836	65.3	674	60.6
Belief in a god-giving life meaning								
Agree	482	45.5	1,137	84.0	112	8.7	137	12.3
Neither agree nor disagree	214	20.2	113	8.4	110	8.6	134	12.1
Disagree	364	34.3	103	7.6	1,059	82.7	841	75.6
Belief in afterlife (yes)	846	79.8	1,252	92.5	400	31.2	402	36.2
Belief in heaven (yes)	860	81.1	1,294	95.6	287	22.4	309	27.8
Belief in hell (yes)	765	72.2	1,290	95.3	137	10.7	248	22.3
Belief in miracles (yes)	813	76.7	1,125	83.1	230	18.0	322	29.0
Happiness (very happy)	482	45.5	221	16.3	224	17.5	209	18.8
SRH (very good)	509	48.0	650	48.0	799	62.4	403	36.2

### Analysis

Data analysis was conducted using IBM SPSS version 25 (IBM, 2017). Missing values were handled by deleting incomplete cases, since less than 5% of values were missing in each country dataset, and missing value analysis showed non-response could reasonably be assumed to be missing at random. Multivariate analyses were conducted using binary logistic regression to examine the relationship between levels of happiness and SRH and R/S beliefs while controlling for the effect of covariates. Prior to analysis, all assumptions were tested in each country dataset.

Logistic regression techniques are widely used in the social sciences and are generally considered as the best choice when the outcome variables of interest are dichotomous or categorical (Osborne, 2015). Moreover, most of the independent variables in this study are also categorical, considering that single Likert items have been used, and these should not be treated as interval data (Carifio & Perla, 2008). Binary logistic regression allows the calculation of the odds or probability of a case belonging to one category or the other in a binary outcome, predicted by a combination of categorical and continuous independent variables (Field, 2018). This type of regression technique requires fewer assumptions than linear or multiple regression. For example, binary logistic regression does not assume a linear relationship between predictor and outcome variables (Tabachnick & Fidell, 2014). This is an important point for this study because research has suggested that the relationship between R/S and SWB may not be linear (Galen & Kloet, 2011).

Other variations of logistic regression were also explored. Since the outcome variables of happiness and SRH had four and five categories, respectively, multinomial regression models were attempted (recoding each outcome in three categories). However, this option was abandoned as it increased the amount if cells with zero frequencies, and also was a less parsimonious approach considering that models were repeated several times due to having two outcomes and four countries for analysis. A second variation explored for data analysis was ordinal logistic regression (also with outcome variables recoded in three categories). This extension of logistic regression takes advantage of the fact that the outcome variable is ordinal and describes the effect of each predictor variable in terms of the probabilities of a case ascending or descending in order. The data, however, did not meet the assumption of ‘proportional odds’ (Osborne, 2015) and therefore binary logistic models were used for each country to facilitate comparative interpretation of results. The binary logistic regression models consisted of seven covariates (the six demographic control variables and service attendance), and six R/S beliefs. Variables were entered hierarchically, with the first block containing the covariates and the second block introducing the six R/S beliefs. This model was replicated in every country sample, for both SWB outcomes. Odds ratios (OR) and 95% confidence intervals (CI) are presented.

## Results

Table 3 shows the odds ratios and 95% confident intervals for each R/S belief (and demographic variable), per country, for the happiness outcome. R/S beliefs made a statistically significant contribution to the prediction of happiness in the Turkish sample, but did not appear to be significantly associated with happiness in the secular countries of Denmark and Czech Republic, and neither in the USA.

**Table 3:** Adjusted odd ratios for demographics and R/S beliefs as predictors of happiness across countries

Predictor variable	USA^a^		Turkey^b^		Denmark^c^		Czech Republic^d^	
	OR	(95% CI)	OR	(95% CI)	OR	(95% CI)	OR	(95% CI)
Demographics								
Female sex	1.05	(0.80, 1.37)	1.00	(0.70, 1.43)	0.91	(0.66, 1.24)	1.44*	(1.04, 2.01)
Age group (ref. ≤ 37 years)								
Middle-aged adults (38 to 57)	0.95	(0.69, 1.31)	0.87	(0.62, 1.24)	0.58**	(0.39, 0.85)	0.39***	(0.26, 0.60)
Older adults (58 +)	1.03	(0.72, 1.46)	1.02	(0.58, 1.78)	0.48**	(0.31, 0.75)	0.23***	(0.15, 0.36)
Post-secondary education (ref. secondary or less)	1.09	(0.82, 1.44)	0.73	(0.48, 1.11)	1.34	(0.96, 1.88)	1.69**	(1.18, 2.44)
Employed (ref. not working)	1.07	(0.80, 1.43)	0.93	(0.64, 1.36)	0.95	(0.65, 1.39)	1.11	(0.75, 1.64)
Married/partnered (ref. not married)	2.53***	(1.95, 3.30)	1.26	(0.90, 1.77)	1.55*	(1.10, 2.19)	1.54*	(1.10, 2.15)
Top placement in society (ref. middle/bottom)	1.79***	(1.31, 2.44)	2.24***	(1.62, 3.10)	2.54***	(1.86, 3.47)	1.80*	(1.11, 2.91)
Service attendance (ref. never)								
Irregular	0.97	(0.69, 1.37)	1.14	(0.63, 2.06)	1.27	(0.91, 1.79)	0.85	(0.57, 1.28)
Regular	1.38	(0.95, 2.01)	1.19	(0.70, 2.04)	1.73	(0.75, 4.00)	1.18	(0.57, 2.42)
R/S beliefs								
Concerned God (ref. Disagree)								
Neither agree nor disagree	1.32	(0.76, 2.28)	0.43	(0.17, 1.10)	0.92	(0.54, 1.55)	0.76	(0.45, 1.27)
Agree	1.12	(0.65, 1.93)	0.50**	(0.30, 0.83)	1.54	(0.85, 2.77)	1.04	(0.55, 1.97)
God-giving life meaning (ref. Disagree)								
Neither agree nor disagree	1.23	(0.83, 1.81)	1.74	(0.84, 3.59)	0.76	(0.39, 1.48)	0.63	(0.32, 1.21)
Agree	1.51*	(1.05, 2.18)	0.86	(0.46, 1.62)	1.75	(0.83, 3.69)	1.33	(0.70, 2.56)
Belief in afterlife (ref. No)	1.36	(0.87, 2.12)	0.23***	(0.12, 0.46)	0.88	(0.57, 1.36)	0.85	(0.54, 1.32)
Belief in heaven (ref. No)	1.23	(0.64, 2.35)	12.14**	(2.68, 55.08)	1.20	(0.67, 2.14)	1.21	(0.66, 2.19)
Belief in hell (ref. No)	0.81	(0.52, 1.26)	0.37	(0.11, 1.26)	0.56	(0.28, 1.11)	1.01	(0.58, 1.74)
Belief in miracles (ref. No)	0.97	(0.62, 1.52)	1.75*	(1.04, 2.94)	0.80	(0.47, 1.37)	1.12	(0.70, 1.79)
*R*^2^ Cox and Snell	0.097		0.051		0.057		0.081	
*R*^2^ Nagelkerke	0.130		0.086		0.094		0.131	
Second block	*X*^2^(8) = 14.137		*X*^2^(8) = 43.701***		*X*^2^(8) = 10.851		*X*^2^(8) = 9.064	
Model	*X*^2^(17) = 108.052***		*X*^2^(17) = 70.555***		*X*^2^(17) = 74.743***		*X*^2^(17) = 94.241***	

^a^*n* = 1060. ^b^*n* = 1353. ^c^*n* = 1281. ^d^*n* = 1112

*p < 0 .05, **p < 0.01, ***p < 0.001

In the Turkish sample, the addition of R/S beliefs in the second block [*χ*^2^(8) = 43.70, *p* < 0.001] made a statistically significant contribution to the final model [*χ*^2^(17) = 70.56, *p* < 0.001, Hosmer and Lemeshow, *p* = 0.441], with an increase in pseudo-*R*^2^ values between the first and the second blocks (Nagelkerke *R*^2^ rose from 0.033 to 0.086). The R/S belief exerting the strongest effect in the prediction of happiness was belief in heaven [OR (95% CI) 12.14 (2.68, 55.08), *p* = 0.001], indicating that believers were about 12 times more likely to be in the ‘very happy’ group compared to non-believers. Turkish participants who believed in miracles were also 75% more likely to be in the ‘very happy’ than ‘fairly happy or less’ group [OR (95% CI) 1.75 (1.04, 2.94), *p* = 0.036] when compared to those not believing in miracles. Other R/S beliefs in the Turkish sample had the opposite effect: those who believed in the afterlife were more than four times less likely to be in the ‘very happy’ group compared to non-believers [OR (95% CI) 0.23 (0.12, 0.46), *p* < 0.001]. Similarly, Turkish participants who endorsed the belief of a God concerned with humans personally were half less likely to report being very happy [OR (95% CI) 0.50 (0.30, 0.83), *p* = 0.007].

For the USA, only belief in ‘God-giving life meaning’ was statistically significantly associated with happiness [OR (95% CI) 1.51 (1.05, 2.18), *p* = 0.028] suggesting that those who held this belief were about 50% more likely to be in the ‘very happy’ category compared to those who did not believe. However, this was not enough to increase the statistical significance of the model already achieved from the demographic variables. No statistically significant associations were found between any of the R/S beliefs and the happiness outcome in the secular countries of Denmark and Czech Republic.

The binary logistic regression model was repeated for the SRH outcome (Table 4). R/S beliefs were only associated with SRH in the Turkish sample, but not in the samples for the USA, Denmark, or Czech Republic.

**Table 4:** Adjusted odd ratios for demographics and r/s beliefs as predictors of SRH across countries

Predictor variable	USA^a^		Turkey^b^		Denmark^c^		Czech Republic^d^	
	OR	(95% CI)	OR	(95% CI)	OR	(95% CI)	OR	(95% CI)
Demographics								
Female sex	0.91	(0.70, 1.19)	0.95	(0.73, 1.24)	1.05	(0.81, 1.34)	1.34*	(1.02, 1.78)
Age group (ref. ≤ 37 years)								
Middle-aged adults (38 to 57)	0.80	(0.58, 1.10)	0.55***	(0.42, 0.70)	0.40***	(0.29, 0.55)	0.37***	(0.26, 0.54)
Older adults (58 +)	0.84	(0.60, 1.19)	0.39***	(0.25, 0.62)	0.45***	(0.31, 0.64)	0.16***	(0.11, 0.23)
Post-secondary education (ref. secondary or less)	2.16***	(1.64, 2.85)	1.26	(0.94, 1.68)	1.31*	(1.02, 1.69)	2.05***	(1.47, 2.85)
Employed (ref. not working)	1.62**	(1.22, 2.17)	1.42*	(1.07, 1.87)	1.81***	(1.35, 2.44)	1.31	(0.94, 1.82)
Married/partnered (ref. not married)	1.23	(0.94, 1.59)	1.13	(0.88, 1.45)	1.32*	(1.01, 1.73)	1.12	(0.84, 1.49)
Top placement in society (ref. middle/bottom)	1.54**	(1.12, 2.10)	1.77***	(1.36, 2.31)	2.59***	(1.93, 3.47)	2.66***	(1.65, 4.27)
Service attendance (ref. never)								
Irregular	1.17	(0.83, 1.64)	0.78	(0.51, 1.21)	1.57***	(1.20, 2.06)	0.72	(0.51, 1.01)
Regular	1.97***	(1.35, 2.88)	0.69	(0.46, 1.01)	1.57	(0.72, 3.42)	1.06	(0.56, 2.00)
R/S beliefs								
Concerned god (ref. Disagree)								
Neither agree nor disagree	1.06	(0.62, 1.81)	1.45	(0.71, 2.98)	0.81	(0.55, 1.20)	0.72	(0.47, 1.11)
Agree	0.78	(0.46, 1.34)	1.72*	(1.09, 2.72)	0.81	(0.49, 1.34)	1.02	(0.58, 1.80)
God-giving life meaning (ref. disagree)								
Neither agree nor disagree	0.87	(0.59, 1.28)	0.89	(0.48, 1.64)	0.81	(0.49, 1.35)	1.00	(0.60, 1.67)
Agree	0.74	(0.51, 1.07)	0.61	(0.36, 1.01)	0.94	(0.49, 1.81)	1.17	(0.66, 2.08)
Belief in afterlife (ref. No)	1.19	(0.77, 1.84)	1.02	(0.56, 1.85)	0.85	(0.60, 1.20)	1.38	(0.94, 2.03)
Belief in heaven (ref. No)	1.38	(0.72, 2.61)	2.64	(0.85, 8.19)	1.35	(0.84, 2.18)	0.62	(0.37, 1.05)
Belief in hell (ref. No)	0.73	(0.47, 1.14)	0.81	(0.28, 2.31)	0.98	(0.57, 1.69)	0.92	(0.57, 1.50)
Belief in miracles (ref. No)	1.07	(0.69, 1.66)	1.04	(0.73, 1.48)	1.23	(0.80, 1.89)	1.09	(0.72, 1.63)
* R*^2^ Cox and Snell	0.093		0.069		0.098		0.176	
* R*^2^ Nagelkerke	0.124		0.092		0.133		0.241	
Second block	*X*^2^(8) = 10.457		*X*^2^(8) = 17.364*		*X*^2^(8) = 5.534		*X*^2^(8) = 9.025	
Model	*X*^2^(17) = 103.126***		*X*^2^(17) = 96.342***		*X*^2^(17) = 131.425***		*X*^2^(17) = 215.440***	

^a^*n* = 1060. ^b^*n* = 1353. ^c^*n* = 1281. ^d^*n* = 1112

*p < 0.05, **p < 0.01, ***p < 0.001

In the Turkish sample, the only R/S belief that was statistically significantly associated with SRH was agreement with a concerned God, which increased the likelihood of participants reporting to have ‘very good SRH’ by 70% [OR (95% CI) 1.72 (1.09, 2.72), *p* = 0.020], compared to those who did not endorse this belief. This effect made a significant contribution to the final model [*χ*^2^(17) = 96.34, *p* < 0.001, Hosmer and Lemeshow, *p* = 0.169, Nagelkerke *R*^2^ = 0.092].

In the US sample, regular church attendance was statistically significantly associated with SRH, with participants who attended church services regularly being almost twice more likely to report having ‘very good SRH’ [OR (95% CI) 1.97 (1.35, 2.88), *p* < 0.001], compared to those who never attended church. In Denmark, it was irregular church attendance which showed a statistically significant association with SRH. Danish participants who attended church services only on an irregular basis were 57% more likely to report having ‘very good SRH’ [OR (95% CI) 1.57 (1.20, 2.06), *p* < 0.001] in comparison with Danes who did not attend services. In relation to the other covariates, the number of demographic variables reaching statistical significance tended to be higher in the secular countries than in the religious ones, for both outcomes (see Tables 3 and 4).

## Discussion

The results of this study indicated that for the US sample there appeared to be no evidence of a relationship between R/S beliefs and SWB outcomes, with only one small positive association between ‘God-giving life meaning’ and happiness that did not significantly contribute to the model beyond the covariates included. However, in Turkey, R/S beliefs made a significant contribution to the prediction of SWB outcomes over the block with covariates alone. Statistically significant relationships indicated that some beliefs (heaven and miracles) increased the odds of people in the Turkish sample falling in the ‘very happy’ category, whereas other beliefs (afterlife and ‘concerned God’) decreased the likelihood of being in the ‘very happy’ group. In addition, the ‘concerned God’ belief was positively associated with SRH. Regarding the two secular countries, Denmark and Czech Republic, there appeared to be no association between any of the R/S beliefs and either of the SWB outcomes.

One modest positive association was found in the American sample, between happiness and the belief that life is meaningful because of God. However, the relationship was too weak to reflect any improvement in the prediction of happiness over the other variables in the model. These results suggest that in the American sample, the relationships between each R/S belief and SWB, if present, were not independent from each of the other beliefs or from service attendance. A possible reason for this finding could be the characteristics of the American religious landscape itself. It has been argued that religiosity in the USA is underpinned by a culture of religious pluralism, high competition among Christian denominations and churches, and a positive view towards church attendance and involvement in religious communities (Norris & Inglehart, 2011). Regarding church attendance in the USA, Krause and colleagues have argued in several studies that the health benefits obtained from general R/S are linked with religious practice, mutual support among church members, and the fostering of positive psychological traits or virtues in religious congregations (Krause, 2010a; Krause et al., 2015; Krause & Ironson, 2019, Krause et al., 2019b). Indeed, in the current study, regular church attendance was positively associated with SRH in the American sample. It is plausible then, that R/S beliefs were not independently associated with SWB in this study due to the ubiquitous quality of the American sociocultural religious life. Another possibility is that service attendance mediates the relationship between R/S beliefs and SWB in the USA; however, the exploration of this possibility was not within the scope of the present study. Lastly, yet another interpretation could be drawn from research conducted by Cragun et al. (2016). In their study, the authors found that when R/S is conceptualised as belief in and experience of the supernatural, the association between R/S and well-being is non-existent. However, their study was conducted with two relatively small non-random convenience samples.

In contrast to the findings from the US sample, in Turkey, the R/S beliefs of a concerned God, afterlife, heaven, and miracles, all showed independent associations with happiness, although not all beliefs exerted their influence in the same way. Beliefs in heaven and miracles had a positive impact on participants’ happiness, while beliefs in an afterlife and a ‘concerned God’ had the opposite effect, with participants who held these beliefs less likely to report being ‘very happy’. These different effects of R/S beliefs should be understood in the context of Islamic religion, which places a high importance on beliefs regarding afterlife, heaven, and hell. Islamic beliefs posit that the soul, after undergoing a period of transformations, could spend thousands of years before being admitted to heaven (or hell). During this period, those who are destined for paradise will experience this time as happy and pleasant, whereas those who are going to hell will go through horrible trials (Chittick, 1992). It seems plausible that devout Muslims, who share these expectations of what happens once they die, might feel anxious at the prospect of an ambiguous afterlife while awaiting admission to paradise; at the same time, they might find solace in the idea of reaching heaven. Moreover, the image of Allah is usually seen as more strict and punitive than the God of Christianity (Chittick, 1992) and Islamic faith requires Muslims to strictly follow a number of rules. It could be reasonable to think that if one believes that God is concerned and aware of one’s individual behaviours, and these behaviours somehow fall short in any aspect, then negative affect such as anxiety, guilt, or worry may ensue. However, the results from the Turkish sample also showed that the same belief in a ‘concerned God’ was positively linked with the SRH measure, increasing the likelihood that believers evaluated their health as ‘very good’. One could speculate that those adhering to this belief also adhere to certain health prescriptions that Muslims must follow (e.g. not drinking alcohol), which could have positive psychological effects by promoting a healthy lifestyle (El Azayem & Hedayat-Diba, 1994).

In the highly secular countries of Denmark and Czech Republic, none of the R/S beliefs appeared to be independently related to happiness or SRH. The most likely explanation for these results is the low prevalence of these beliefs across the two samples, as well as the high percentages of atheism and agnosticism in these countries. In relation to the lack of R/S beliefs in Danish society, Zuckerman (2020) has described at length the beliefs, values, and worldviews of Danes. Although most Danish people are tax paying members of the Danish National Church, Zuckerman explains, they only see themselves as cultural Christians, and the vast majority do not believe in any of the supernatural tenets of religion. Danish people associate being Christian with their culture, their collective past, and traditional ways of celebrating ceremonies such as marriage. Findings from Storm et al. (2019) based on analysis from national survey data confirm this depiction of ‘civil religion’ or ‘cultural religion’.

In the Czech sample, results suggested no evidence of any associations between R/S beliefs and SWB. The Czech Republic is one of the most secular and least religious countries in the world (Edlund, 2013), with a long history of non-religious culture shaped by events starting with anti-clerical movements in the fifteenth century and the rise of nationalistic and secular trends of nineteenth century elites (Hamplová & Nešpor, 2009; Nešpor, 2004). Given this history and cultural context, the lack of associations between R/S beliefs and SWB in the Czech sample is not surprising.

### A Terror Management Theory Perspective

As explained previously, TMT poses that cultural worldviews provide individuals with order, control, and meaning, that helps them to assuage the potentially paralysing anxiety coming from the realisation of their own mortality (Greenberg et al., 1986). Thus, TMT can be seen as a theory of psychological well-being, since it explains what is necessary for individuals to function properly (participation in a meaningful cultural worldview), and why (to buffer the inescapable anxiety of death) (Juhl, 2019).

Considering the hypothesis presented in the introduction of this study, and the possible scenarios that could support or falsify it, the results suggest that an interpretation through TMT can be mostly supported. The relationship between R/S beliefs and SWB was most evident in Turkey, the most religious country of the four, and no relationships were found in the secular countries of Denmark and Czech Republic.

The results from the US sample, however, are somehow less clear. A TMT interpretation of the findings would suggest that the apparent lack of relationships between R/S beliefs and SWB in the US sample may be related to the importance of religiosity experienced through its social bonding aspects, including participation in religious services. Unsurprisingly, this has been one of the most researched pathways between R/S and SWB and health in general (George et al., 2002; Krause, 2010a, 2010b), and might help to explain the lack of evidence for an association between R/S beliefs and SWB when controlling for service attendance.

In the case of Turkey, more particularly, the mix of positive and negative influences of R/S beliefs on happiness may seem to contradict TMT at first: why, if the purpose of a worldview is to buffer the anxiety created by death, would people subscribe to a worldview that soothes anxiety on the one hand, but has the potential to increase it on the other? This seeming contradiction is actually required for an effective anxiety buffering system. The overall conception of the worldview must incorporate positive as well as negative aspects to make it more ‘believable’ (Soenke et al., 2013). Thinking that there is a ‘concerned God’ that may punish behaviour, or that one might face suffering in the afterlife, is the other side of the coin of miracles, heaven, and the general positive implications of supernatural R/S beliefs.

In societies where R/S beliefs and R/S in general are low, as is the case in Denmark and the Czech Republic, the widespread of non-religious, secular worldviews may explain the lack of relationships between R/S beliefs and SWB, as individuals in these societies might be more likely to adhere to other beliefs and employ other mechanisms to buffer death anxiety, according to TMT.

### Study Strengths and Limitations

The findings of the present study should be understood in the context of the following limitations. Although the sample sizes were large for the four countries, the number of people holding (or not holding) specific combinations of beliefs in each sample may be small. As there were several categorical variables included in the study, results of the statistical analyses are likely to include cells with zero frequencies at some of the different subpopulation levels of the dependent variables. This might have affected the standard errors, leading to wider confidence intervals. However, this problem of incomplete information is common, and when several variables are included in a model, practically inevitable (Field, 2018). Furthermore, this study did not address interactions, for example between R/S beliefs and service attendance, or among R/S beliefs themselves.

Notwithstanding these limitations, this study has several strengths. First, this study addressed an important gap of research in the field of R/S and health, focusing on the specific characteristic of R/S, that is supernatural beliefs, instead of the much-theorised pathways that could be understood as secular in nature. The combination of a cross-national design and the replication of identical logistic regression models across countries allowed us to explore the associations between R/S beliefs and SWB in different cultures, and to elucidate whether any purported relationship between these variables could be attributed to some universal religious pattern or to specific cultural contexts.

## Conclusion

The results of this study stress the importance of considering the cultural context when studying relationships between R/S beliefs and SWB, and R/S and well-being and health in general. The importance of religiosity/spirituality in relation to subjective well-being appears to be linked to the type of sociocultural worldview that people engage with in their lives, rather than being some universal phenomenon.

## Data Availability

Data and material provided at request. Please contact the main author.
